# Cooperation between CD4+ T Cells and Humoral Immunity Is Critical for Protection against Dengue Using a DNA Vaccine Based on the NS1 Antigen

**DOI:** 10.1371/journal.pntd.0004277

**Published:** 2015-12-09

**Authors:** Antônio J. S. Gonçalves, Edson R. A. Oliveira, Simone M. Costa, Marciano V. Paes, Juliana F. A. Silva, Adriana S. Azevedo, Marcio Mantuano-Barradas, Ana Cristina M. A. Nogueira, Cecília J. Almeida, Ada M. B. Alves

**Affiliations:** 1 Laboratory of Biotechnology and Physiology of Viral Infections, Oswaldo Cruz Institute, Oswaldo Cruz Foundation, Rio de Janeiro, Brazil; 2 Laboratory of Immunopharmacology, Oswaldo Cruz Institute, Oswaldo Cruz Foundation, Rio de Janeiro, Brazil; University of California, Berkeley, UNITED STATES

## Abstract

Dengue virus (DENV) is spread through most tropical and subtropical areas of the world and represents a serious public health problem. At present, the control of dengue disease is mainly hampered by the absence of antivirals or a vaccine, which results in an estimated half worldwide population at risk of infection. The immune response against DENV is not yet fully understood and a better knowledge of it is now recognized as one of the main challenge for vaccine development. In previous studies, we reported that a DNA vaccine containing the signal peptide sequence from the human tissue plasminogen activator (t-PA) fused to the DENV2 NS1 gene (pcTPANS1) induced protection against dengue in mice. In the present work, we aimed to elucidate the contribution of cellular and humoral responses elicited by this vaccine candidate for protective immunity. We observed that pcTPANS1 exerts a robust protection against dengue, inducing considerable levels of anti-NS1 antibodies and T cell responses. Passive immunization with anti-NS1 antibodies conferred partial protection in mice infected with low virus load (4 LD_50_), which was abrogated with the increase of viral dose (40 LD_50_). The pcTPANS1 also induced activation of CD4^+^ and CD8^+^ T cells. We detected production of IFN-γ and a cytotoxic activity by CD8^+^ T lymphocytes induced by this vaccine, although its contribution in the protection was not so evident when compared to CD4^+^ cells. Depletion of CD4^+^ cells in immunized mice completely abolished protection. Furthermore, transfer experiments revealed that animals receiving CD4^+^ T cells combined with anti-NS1 antiserum, both obtained from vaccinated mice, survived virus infection with survival rates not significantly different from pcTPANS1-immunized animals. Taken together, results showed that the protective immune response induced by the expression of NS1 antigen mediated by the pcTPANS1 requires a cooperation between CD4^+^ T cells and the humoral immunity.

## Author Summary

Dengue is an emerging mosquito-borne disease present in an extensive area of the globe with an estimated risk exposure of half of the world’s population. Unfortunately, no specific treatment or vaccine is available to control this disease, which leads to approximately 20,000 casualties annually. The protective immune response against this pathogen consists of an important goal for the development of anti-dengue strategies. For years, the presence of neutralizing antibodies was believed to represent the major response for protection against dengue. However, a recent clinical trial showed that despite the induction of a balanced antibody response against all serotypes, vaccination had only a partial efficacy. In the present work, we aimed to elucidate the contribution of the cellular and humoral responses elicited by a DNA vaccine candidate encoding the non-structural 1 protein (NS1) from dengue virus. We observed that antibody as well as T cell responses are important for protection against dengue in a cooperative way. Our results demonstrated that an effective defense against virus was not achieved with antibodies or T cells alone, but rather with the combination of both responses. Therefore, we suggest that an ideal vaccine against dengue should induce both arms of the immune system.

## Introduction

Dengue represents the most important human mosquito-borne disease worldwide. Each year, an estimated 96 million people present clinical signs of the disease [1], resulting in about 20000 deaths [2]. The illness is caused by the dengue virus (DENV), which consists of four distinct serotypes (DENV1-4) present in tropical and subtropical regions of the globe. Infection may be asymptomatic or can be manifested as a non-differentiate febrile, marked mainly by myalgia, headache and retroorbital pain. The most severe forms of the disease are characterized by plasma leakage, thrombocytopenia and hemorrhage, which can evolve to hypovolemic shock [3–4].

The DENV genome is a single positive RNA strand of approximately 11 kb, which is translated into a single polyprotein. This polyprotein is further cleaved into three structural proteins, capsid (C), premembrane (prM), and envelope (E), and seven nonstructural proteins (NS1, NS2A, NS2B, NS3, NS4A, NS4B, and NS5) [5].

The NS1 is a conserved N-linked glycoprotein, which is synthesized as a monomer and dimerizes after post translational modification in the lumen of the endoplasmic reticulum [5]. This glycoprotein is found in mammalian infected cells associated with plasma membrane and also secreted into the circulation as a soluble multimer, with reports of up to 50 μg/mL in the serum of some dengue patients [6–11]. The NS1 is still an enigmatic protein in which the mechanistic function remains somewhat unknown. Intracellular NS1 of many flavivirus co-localizes with dsRNA and other components of the viral replication complex and plays an essential role in replication [12–16]. The secreted form of NS1, in its turn, seems to be implicated in immune evasion strategies. It may inhibit, for instance, complement activation by binding to the regulatory protein factor H [17]. The NS1 is highly immunogenic, inducing significant levels of anti-NS1 antibodies in dengue infected patients [18–20]. Some reports have pointed the NS1 as a target antigen for the development of dengue vaccines [21–26], while others suggested a role for this protein in the pathogenesis [27–34]. Thus, it still remains an apparent paradox due to its ability to elicit both protective and potentially pathogenic immune responses.

No currently antiviral treatment against dengue is available and the development of an effective anti-dengue vaccine would represent a cornerstone in public health. An important aspect of dengue is that an effective immunity can be potentially impaired during heterologous infections, which may lead to severe manifestations of dengue and represents a great burden in the development of a vaccine against this pathogen [35–40]. Therefore, there is a consensus that a vaccine against dengue should be tetravalent, inducing a long term protective immunity. In this environment, a better understanding of the immunological mechanisms by which a protective immunity against dengue is generated became critical for the development of a vaccine. For years, the presence of serum neutralizing antibodies was believed to represent the major component of an effective protection against the infection. Yet, a recent clinical trial showed that neutralizing antibodies alone might not constitute the only key element to confer protection. In fact, despite of a balanced antibody response against all serotypes in this phase IIb trial, vaccination resulted in partial efficacy [41].

In this context, we analyzed herein the protective immune response elicited by a DNA vaccine (pcTPANS1) encoding the NS1, which, as a non-structural protein, does not elicit neutralizing antibodies. We have previously reported that this DNA vaccine can be protective against DENV infection in mice [23,24]. In the present work, we showed that this protection is robust and in part characterized by anti-NS1 specific antibodies and T cells responses. We found that cooperation between CD4^+^ T cells and the humoral response plays a critical role on the protection against dengue mediated by the NS1 antigen. Our data provides new insights on the immunity elicited by DENV NS1 antigen as well as on a prospect for vaccine development.

## Materials and Methods

### DNA vaccine

Immunizations were performed using a DNA vaccine, pcTPANS1, previously described [23,24]. Briefly, this plasmid, derived from pcDNA3 (Invitrogen, USA), encodes the full length NS1 gene from DENV2, strain New Guinea C, fused to the human tissue plasminogen activator signal sequence (t-PA). The pcTPA plasmid [22], without the NS1 gene, was used as control. Plasmids were isolated from transformed *Escherichia coli*, DH5-α strain, and purified by Qiagen Endofree Plasmid Giga Kit (Qiagen, Germany) following manufacturer’s instruction. Purified plasmids were eluted in endotoxin-free sterile water and kept at -20°C until use.

### Ethics statement

The study in mice was carried out in accordance with ethical principles in animal experimentation stated in the Brazilian College of Animal Experimentation and approved by the Oswaldo Cruz Institute’s Animal Use Ethical Committee (approval ID: L067/08 and LW14/12).

### DNA immunization

Wild-type SPF male Balb/c mice, 4 to 6 week-old, were purchased from the Multidisciplinary Center for Biological Investigations (CEMIB, UNICAMP-SP). Animals were inoculated by the intramuscular (i.m.) route with 50 μg of plasmids diluted in 50 μL of phosphate buffer saline (PBS) in each tibialis posterior muscles (100 μg/mice) using 27-gauge needles. Each animal group received two doses of the recombinant plasmid, pcTPANS1, or control vector, pcTPA, given 2 weeks apart. Cells and/or sera were collected four weeks after the first immunization or 21 days after virus challenge.

### Histological analysis

Liver tissue samples from Balb/c mice immunized with pcTPANS1 or naïve animals were fixed in formalin (10%), blocked in paraffin resin, cut in 4μm, deparafinized in xylene and rehydrated with alcohol, as described elsewhere [42]. Sections were stained with hematoxylin and eosin for histological examination and visualized in a Nikon ECLIPSE E600 microscope.

### Quantification of hepatic enzymes in serum samples

Levels of alanine aminotransferase (ALT) and aspartate aminotransferase (AST) were measured (U/L) in serum samples of BALB/c mice inoculated with pcTPANS1 or pcTPA. Animals were bled 4 weeks after DNA injection and enzymes were quantified by the biochemical analyzer Reflotron Plus (Roche, Switzerland) as determined by the manufacturer.

### Virus challenge

Animals were challenged by the intracerebral (i.c.) route with a mouse brain adapted DENV2, strain New Guinea C (GenBank M29095). Mice were anesthetized with a mixture of ketamine-xylazine [43] and inoculated with 30 μL of DENV2 suspensions, corresponding to 4 or 40 LD_50_, diluted in E199 medium supplemented with 5% fetal bovine serum (FBS, Invitrogen). Animals were monitored for 21 or 40 days.

### Detection of viremia

Serum samples, obtained from naïve or pcTPANS1-vaccinated mice challenged with DENV2, were collected 9 days after virus infection. Virus was detected by plaque assay in Vero cell monolayers. Cells were grown in 24-well plates with medium E199, 1% garamycin, buffered with 5% sodium bicarbonate, supplemented with 5% FBS, and maintained at 37°C in 5% CO2. On the next day, serum samples were added to cell monolayers, followed by incubation for 1h at 37°C in 5% CO2. Culture medium was then removed and cells were maintained for 6 days with 1ml of semi-solid E199 medium (with 3% carboxymethylcellulose, 1% garamycin, buffered with 5% sodium bicarbonate, supplemented with 5% FBS) also at 37°C in 5% CO2. After this period, cells were fixed with 10% formalin, stained with crystal violet and plaques were manually counted. Negative control was performed with sera from non-immunized mice.

### Detection of anti-NS1 antibody response

Mouse serum samples were tested (individually or pooled) for the presence of NS1-specific antibodies by ELISA. Briefly, MaxiSorp plates (Nunc, Denmark) were coated with 0.4μg / well of refolded recombinant NS1 protein [44] in PBS, and incubated for 1 h at 37°C. After this period, wells were overnight-blocked with 2% skim milk in 0.05% Tween-20-PBS (PBST). On the next day, serum samples were serially diluted and added to plates previously washed 5 times with PBST. After 1 h at 37°C, plates were washed again with PBST and incubated with goat anti-mouse IgG conjugated with horseradish peroxidase (Southern Biotechnology, USA) for 1 h at 37°C. Plates were washed in PBST and incubated with ortho-phenylenediamine dihydrochloride (Sigma, USA) and H_2_O_2_ for 20 min at room temperature. Reaction was stopped with 9N H_2_SO_4_ solution and visualized at A 490 nm. Titers were established as the reciprocal of serum dilution, which gave absorbance higher than mean values of respective non-immunized mouse samples.

### Serum transfer

Groups of pcTPA- or pcTPANS1-immunized mice were anesthetized with ketamine-xylazine and sacrificed 15 days after the second DNA dose. Animals were bled by cardiac puncture and sera were pooled and kept at -70°C until use. For passive immunization by antibody transfer, mice were inoculated intraperitoneally (i.p.) with 300 μL of these sera, three hours before virus injection and every three days after challenge (total of 7 inoculations). Antibody transfer was also performed simultaneously with adoptive cell transfer experiments. In this case, animals were i.p. inoculated with one dose of 500 μL of pcTPANS1-immunized mouse serum before virus challenge. Animals were followed up for 21 days to determine survival rates.

### Flow cytometry

Splenocytes from Balb/c mice were isolated and erythrocytes were lysed by treatment with FACS lysing solution (BD Biosciences, USA), prepared according to manufacturer’s instructions. Cells were washed in PBS and suspended in 1% (w/v) bovine serum albumin (Sigma) prepared in PBS. Approximately 10^6^ splenocytes were pelleted and stained for 30 min at 4°C in the dark with 20 μL of fluorescent monoclonal antibodies against: CD3-PE, CD4-FITC or CD4-Alexa Fluor 647, CD8-PerCP, B220-APC and CD45RB-FITC (BD Biosciences), previously titrated and mixed. Splenocytes were washed twice, suspended in 300 μL of PBS and read in a BD Accuri C6 Flow Cytometer (BD, USA). Cell populations were analyzed offline using FlowJo software (Tree Star, USA). For the *in vivo* cytotoxicity analysis, CFSE-stained cells were readily analyzed without additional markers.

### Interferon-γ ELISPOT assay

Splenocytes from pcTPA- or pcTPANS1-immunized mice (n = 5) were isolated 15 days after the last immunization and used in IFN-γ ELISPOT test. Cells were isolated as described above and suspended in RPMI-1640 medium (Sigma) with gentamicin (0.04 mg/ mL, Sigma). The assay was performed with a synthetic peptide (^265^AGPWHLGKL^273^) present in the NS1 protein of DENV2, described as specific for CD8^+^ T cells [45]. The IFN-γ ELISPOT mouse set (BD Biosciences) was used in accordance to the manufacturer’s instruction. Briefly, 96-well plates were coated overnight at 4°C with 5 μg/mL IFN-γ capture monoclonal antibody in PBS, followed by washing and blocking with supplemented RPMI-1640 medium at room temperature. Splenocytes (10^6^ cells/well) were added to plates concomitant with the NS1 peptide in 200 μL of RPMI-1640 medium supplemented with 10% FBS (peptide final concentration of 10 μg/mL). Non-stimulated and concanavalin A (Con A, 5 μg/mL) stimulated cells were used as negative and positive controls, respectively. Splenocytes were cultured for 20 h at 37°C in 5% CO_2_. Plates were washed, followed by incubation with biotinylated IFN-γ detection antibody (2 μg/mL) in PBS with 10% FBS. Plates were then washed with PBST and incubated with streptavidin horseradish peroxidase diluted 1:100. Spots were revealed with AEC substrate reagent set (BD Bioscience) at room temperature and counted with an Immunospot reader (Cellular Technology Ltd, USA) using the Immunospot Software Version 3. Results were expressed as the average of spot-forming cells (SFC) per 10^6^ cells, from triplicate wells, after subtraction of background values detected in non-stimulated splenocytes.

### *In vivo* citotoxicity assay

The *in vivo* cytotoxicity assay was performed with transfer of NS1-peptide-presenting cells to vaccinated recipient mice, based on a previously described protocol [46]. For target cells, splenocytes from naïve syngeneic mice, isolated as described above (item 2.7), were incubated with either 0.5 or 5 μM of carboxyfluorescein diacetate succinimidyl ester (CFSE, CellTrace, Invitrogen), CFSE^low^ and CFSE^high^, respectively, in PBS at 37°C for 15 min. Cells were washed with RPMI-1640 supplemented with 1% FBS, containing 1% penicillin/streptomycin (10.000 U/mL, Invitrogen). The CFSE^high^ cells were then incubated in the presence of 25 μM of the NS1 peptide (^265^AGPWHLGKL^273^) at 37°C for 40 min, whereas CFSE^low^ cells were incubated in medium only. After labeling and peptide pulsing, both cell populations were washed in medium without FBS and mixed in a proportion of 1:1. Cells were intravenously (i.v.) transferred to pcTPA- or pcTPANS1-inoculated mice, which received 10^7^ cells of each population (CFSE^low^ and CFSE^high^) in a single injection (100 μL) by retro-orbital route. Part of these animals was previously challenged with DENV2 three days before cell transfer. Some animals were also depleted from CD4^+^ or CD8^+^ cells before cell transfer. Recipient mice were sacrificed 20h following cell transfer and splenocytes were isolated for flow cytometric analysis. Percentage of specific lysis was determined as follows: Cell lysis (%) = (1—CFSE^high^/CFSE^low^) x 100.

### Depletion of CD4^+^ and CD8^+^ cells

Mice were depleted from CD4^+^or/and CD8^+^T cells by inoculations of in-house produced ascitic fluids before and after virus challenge. Briefly, the in-house ascitic fluids were produced after i.p. inoculation of GK1.5 or 53–6.7 hybridomas (10^7^ cells / animal) in nude Balb/c mice in order to obtain anti-CD4 or anti-CD8 antibodies, respectively. Ascitic fluids were collected approximately 15 days after hybridoma inoculations, centrifuged at 500g for 15 min at 4°C, and supernatants were aliquoted and stored at -70°C. For depletions, vaccinated Balb/c mice were i.p. inoculated with 25 μL of ascitic fluids in days -5, -3 and -1 prior to virus challenge. An additional dose of ascitic fluids was also administered 15 days after virus challenge to ensure cell removal. Non-immunized animals were also depleted from CD4^+^ or CD8^+^ cells before virus challenge. Other controls included vaccinated and naïve animals challenged without depletion. Depletions were previously standardized and confirmed by flow cytometry.

### T cell enrichments and adoptive transfer

Spleens were collected from donors (pcTPANS1 or pcTPA-inoculated mice), obtained two weeks after the second DNA dose and without virus challenge, disrupted using wire mesh screens, and splenocytes were isolated in RPMI-1640, containing 1% penicillin/streptomycin (10.000 U/mL, Invitrogen), supplemented with 5% FBS. Cells were incubated in culture medium only for 1h and for another 1h in nylon wool column (previously packed and stabilized with RPMI in 5% CO_2_ atmosphere), both at 37°C. After elution from nylon wool column with RPMI medium, in order to remove B lymphocytes, CD4^+^ and CD8^+^ cells were further purified by negative selection, using a BioMag and cell sorting kit according to manufacturer’s instructions (Bangs Laboratories Inc, USA). Briefly, T cell enriched suspension was incubated with ascitic fluids containing anti-CD4 or anti-CD8 antibodies for 30 min at 4°C, followed by incubation for 20 min with anti-rat IgG magnetic beads at room temperature. Suspensions were then submitted to a magnetic column for negative selection, i.e. to obtain enriched CD4+ or CD8+ cell samples we incubated cell suspension with anti-CD8 or anti-CD4 antibodies, respectively. Finally, cells were collected in culture medium and counted in Neubauer chamber with trypan blue (Invitrogen) staining to assess cell viability.

For T cell transfer to naïve mice, CD4^+^ or CD8^+^ enriched cell suspensions (10^6^ and 5 x 10^5^ cells, respectively) were i.v. injected by retro-orbital route in a final volume of 100 μL. Injections were performed approximately 18h after virus challenge.

### Statistics

Data were analyzed with GraphPad prism software v5.1 (La Jolla, USA) using non-parametric tests. Statistical significance was determined using Mann-Whitney test for the analysis of data obtained in the hepatic enzymes quantification, ELISA, ELISPOT and flow cytometric assays. Survival distributions were evaluated using Log-Rank statistical test. Significant differences were defined with probability values inferior to 0.05 (*p<0.05; ** p<0.01 and ***p<0.001).

## Results

### Protection elicited by pcTPANS1 vaccination

**Fig 1:**
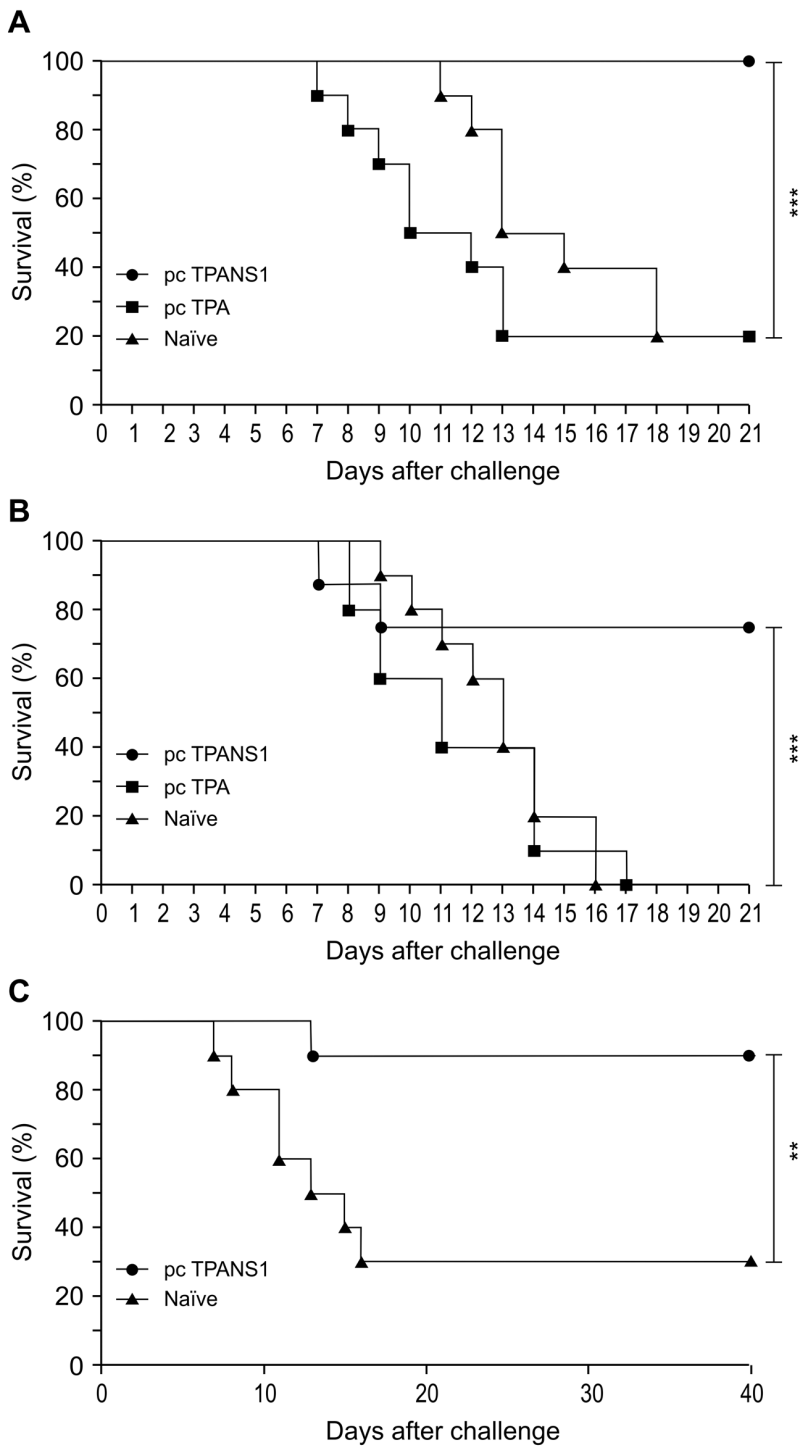
Survival rates of vaccinated or non-immunized Balb/c mice after challenge with DENV2. Groups of Balb/c mice (n = 10) 4 weeks old were inoculated with two doses of the DNA vaccine pcTPANS1 or the control plasmid pcTPA (100 μg of DNA/dose given two weeks apart). (**A and B**) Two or (**C**) four weeks after the second dose, animals were intracerebrally inoculated with (**A**) 4 LD_50_ or (**B and C**) 40 LD_50_ of neuroadapted DENV2 NGC strain. Naïve animals were also challenged with 4 or 40 LD_50_ DENV2. Animals were observed (**A and B**) for 21 or (**C**) 40 days after challenge for evaluation of survival rates. Asterisks indicate significant differences between vaccinated and control animals using Log-Rank statistical test. *** p<0.001,**p<0.01.

The previously constructed pcTPANS1 DNA vaccine contains the DENV2 NS1 gene fused to the t-PA signal sequence, for secretion of the recombinant NS1 protein. After immunization with this DNA vaccine, all Balb/c mice were protected against DENV2 when they were given a viral dose of 4 LD_50_ (Fig 1A). When the viral dose was 10-fold increased (40 LD_50_), we could still observe significant survival rates (approximately 80%) in the vaccinated mouse group when compared to pcTPA-inoculated or naïve control groups, hence suggesting a robust and effective protection induced by the vaccine (Fig 1B). Most of control animals died after virus infection, although approximately 35% of them did not present apparent clinical signs (hind leg paralysis and/or alteration of spinal cord) before death. Viremia was detected by in most of naïve animals inoculated with DENV2 (71%), while only one vaccinated mice presented circulating virus (20%) (S1 Table). Once animals were challenged with DENV2 30 days after immunization, survival rates in pcTPANS1-immunized group were around 90%, indicating a long-term protection conferred by this vaccination (Fig 1C). In addition, survival was followed up to 40 days after virus infection and no death was observed after the first three weeks post challenge (Fig 1C).

**Fig 2:**
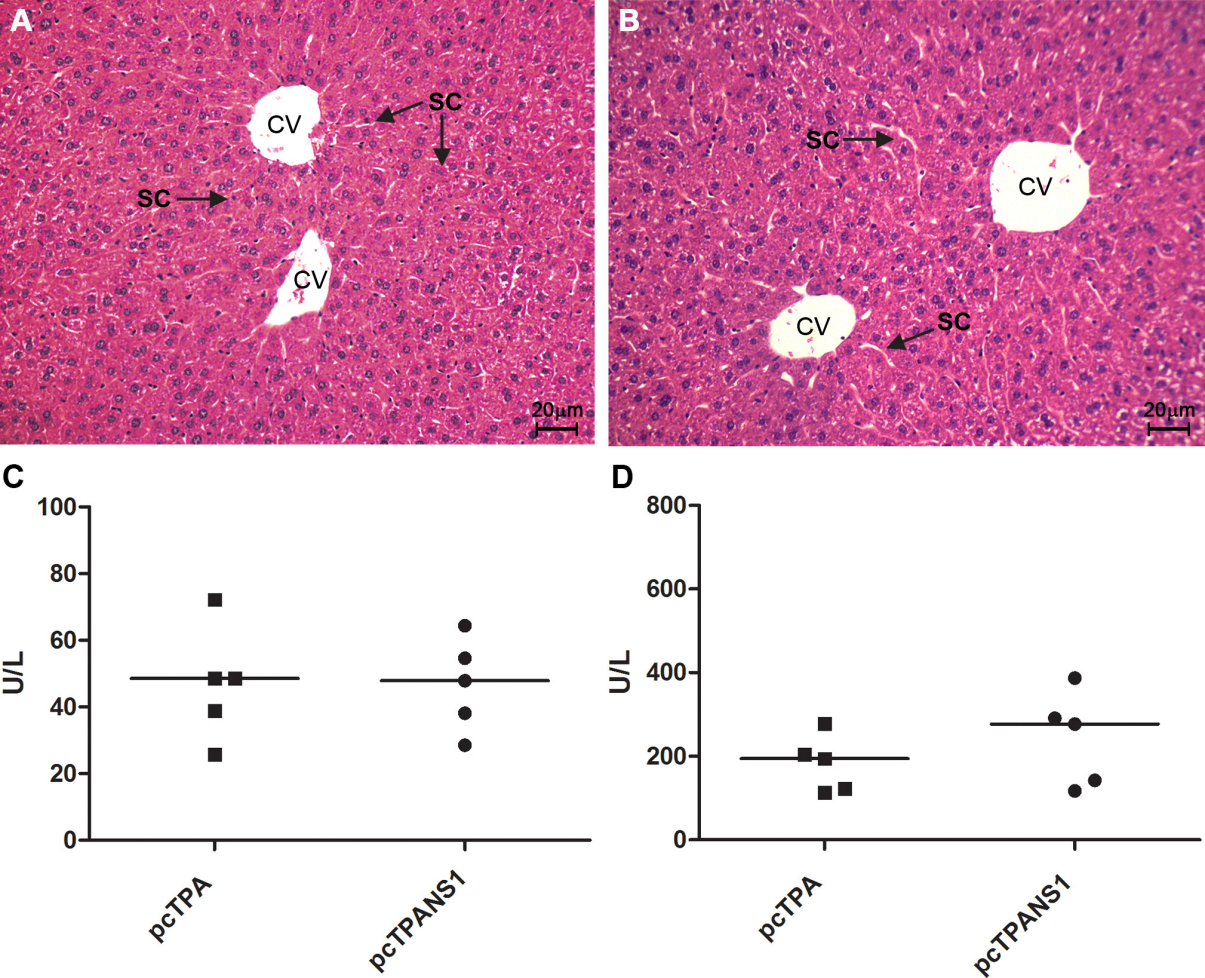
Histological analysis of pcTPANS1-vaccinated mouse liver and quantification of hepatic enzymes. Representative histological analysis of control (naïve) and vaccinated mice stained with H.E. Liver of (**A**) naïve or (**B**) vaccinated mice immunized with two doses of the pcTPANS1 presenting hepatic parenchyma and sinusoidal capillary with regular structure. Central Vein (CV); Sinusoids capillaries (SC). Quantification of **(C)** alanine aminotransferase (ALT) and **(D)** aspartate aminotransferase (AST) in serum samples of pcTPA- and pcTPANS1-inoculated mice (n = 5). Values between both groups were not statistical different when evaluated by Mann-Whitney test.

Previous reports suggested that the NS1 could play a role in inducing hepatic tissue damages. Thus, in order to evaluate whether the NS1 encoded by the pcTPANS1 DNA vaccine generates hepatic injury, we analyzed the liver of immunized animals. Histological analysis of the liver of vaccinated animals revealed a regular structure of the hepatic parenchyma and sinusoidal capillaries, without circulatory alterations or inflammatory infiltrates, similar to what we observed in naïve mice (Fig 2A and 2B). Furthermore, quantification of serum levels of ALT and AST revealed similar values comparing pcTPANS1- and pcTPA-inoculated mice (Fig 2C and 2D), thus confirming the absence of hepatic damages induced by the DNA vaccine.

### Humoral immune response promoted by the pcTPANS1 vaccine

#### Levels of anti-NS1 specific antibodies

**Fig 3:**
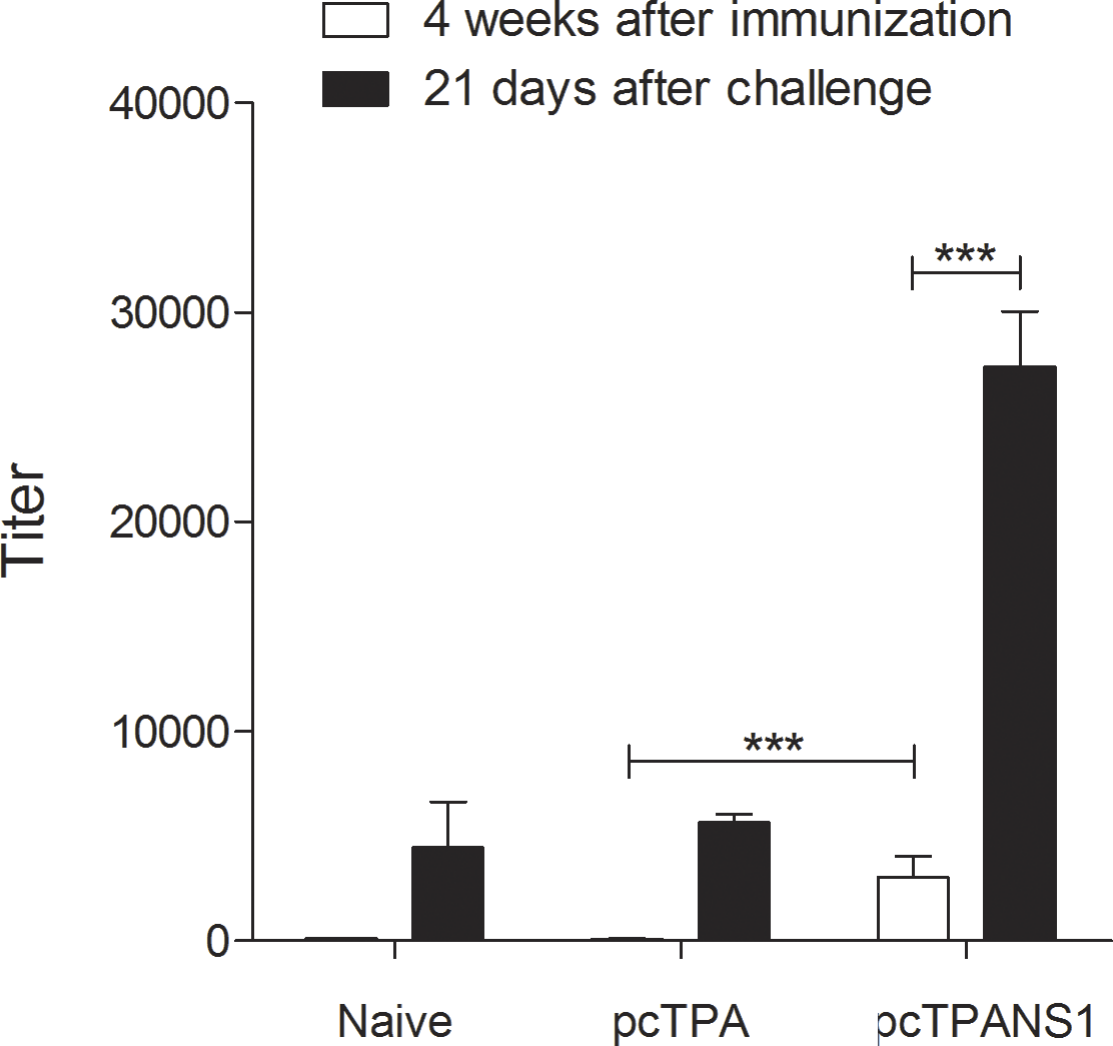
Titration of NS1-specific antibodies. NS1-specific antibodies were quantified in mouse serum samples (n = 5) using recombinant NS1 protein as a solid phase antigen in ELISA plates. Sera were collected before and after DENV2 challenge. Data are represented as mean and standard error of the mean. Asterisks indicate significant differences between groups using Mann-Whitney statistical test. *** p<0.001.

Four weeks after receiving the first DNA dose, only animals vaccinated with the pcTPANS1 presented NS1-specific antibodies, detected by ELISA (Fig 3). Besides, we observed a significant boost of this response 21 days after challenge with DENV2, with NS1-specific antibody titers approximately 9-fold higher than those detected before virus infection. Naïve or pcTPA-inoculated mice that survived virus challenge and presented high morbidity signs also exhibited anti-NS1 antibodies, although in significantly lower levels than those observed in vaccinated animals (Fig 3).

#### Contribution of anti-NS1 humoral response in protection

**Fig 4:**
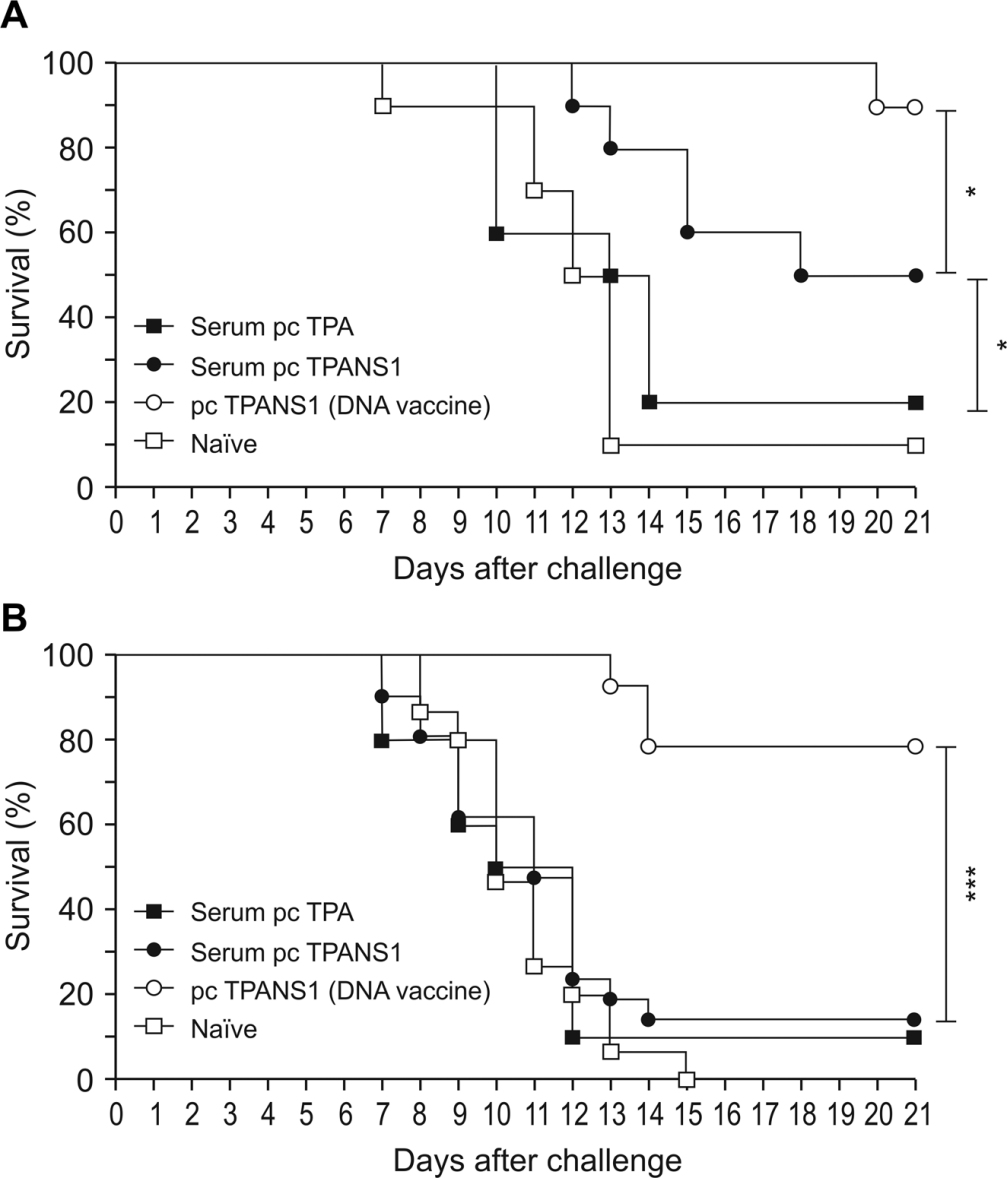
Survival rates of mice passively immunized with anti-NS1 polyclonal antiserum and challenged with DENV2. Balb/c mice (n = 10) were intraperitoneally injected with several doses of anti-NS1 polyclonal antiserum (one dose before and 6 doses after challenge with DENV2). Sera for immunization were obtained from pcTPANS1- or pcTPA-inoculated animals. After challenge with (**A**) 4 LD_50_ or (**B**) 40 LD_50_ mice were monitored for 21 days for the establishment of survival curves. Controls also included pcTPANS1-immunized (DNA vaccine) or naïve mice challenged with DENV2. Arrows indicate time point of serum inoculation. Asterisks indicate significant differences between groups using Log-Rank statistical test. *p<0.05; *** p<0.001.

The protective role of the anti-NS1 antibody was investigated by a passive immunization experiment, where naïve mice were injected with several doses of serum samples collected from pcTPANS1- or pcTPA-inoculated animals. After challenge with DENV2 4 LD_50_, 50% of the animals that received anti-NS1 antiserum (obtained from pcTPANS1-immunized mice) survived virus infection, while 80% and 90% of control mice died (naïve animals or mice injected with serum obtained from animals inoculated with pcTPA plasmid) (Fig 4A). Differences between control animals and mice passively immunized with anti-NS1 antiserum were statistically significant. However, protection conferred by the DNA vaccine was significantly higher than that generated by the serum transfer (Fig 4A). Besides, the partial protection observed by serum transfer was completely abolished when challenge was performed with DENV2 40 LD_50_. In this case, survival rates were similar to control groups (less than 15%), whereas 80% of vaccinated mice remained protected (Fig 4B).

### Activation of the cellular immune response by the pcTPANS1 vaccine

**Fig 5:**
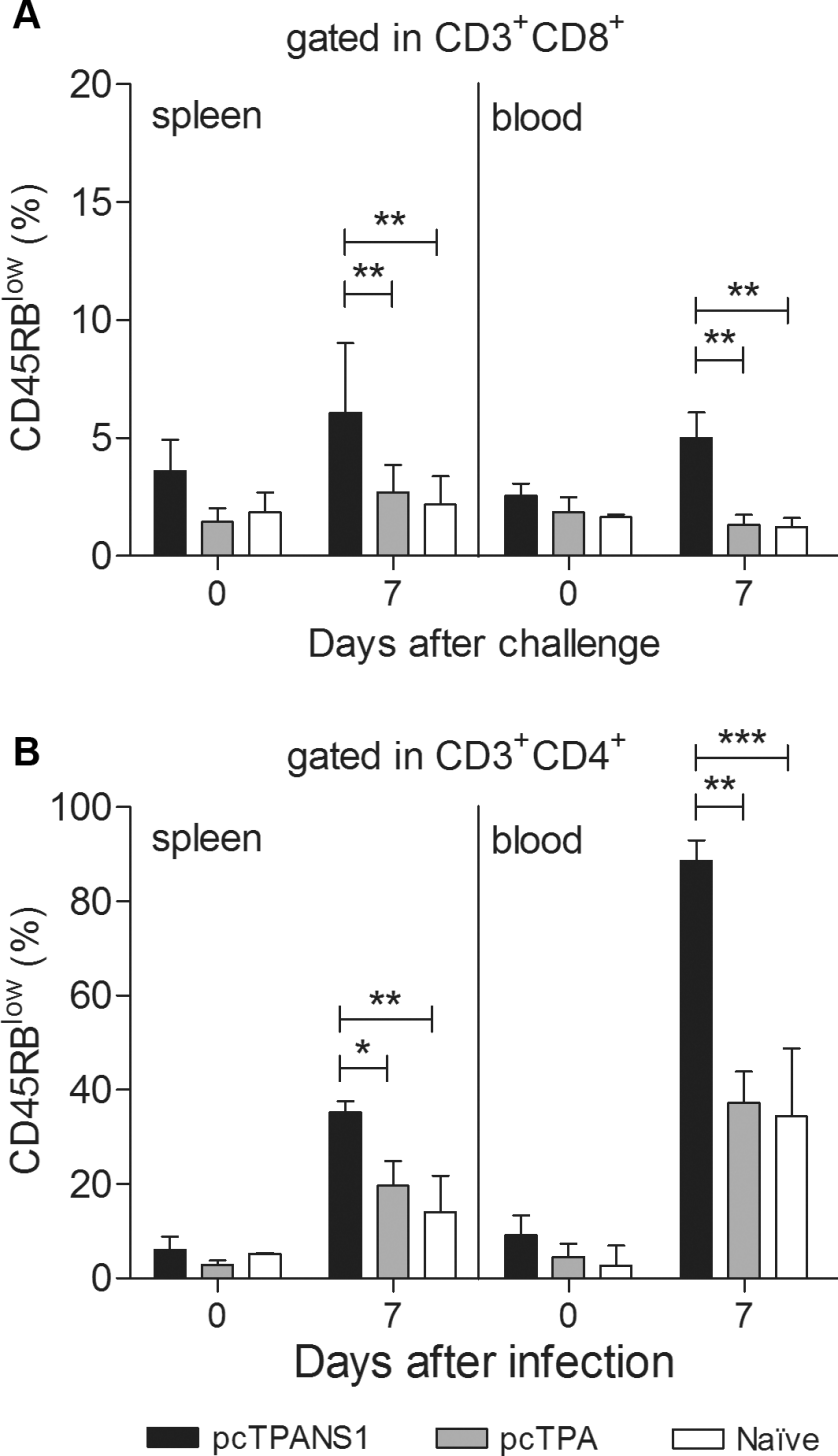
Activation of T cells in vaccinated Balb/c mice challenged with DENV2. Balb/c mice (5 to 10 per group) were previously immunized with pcTPANS1 and further challenged with DENV2. Control groups (non-vaccinated or inoculated with pcTPA plasmid) were also challenged. Spleen and blood samples were collected from animals at days 0 and 7 after infection and prepared for multicolor flow cytometry analysis using anti-CD3, anti-CD4, anti-CD8 and anti-CD45RB. Values are expressed as median and interquartile range of CD45RB^low^cells gated in CD3^+^ cells and **(A)** CD8^+^ or in **(B)** CD4^+^ T cells. Statistical differences between groups were evaluated using Mann-Whitney test (*p<0.05; **p<0.01 and ***p<0.001). Data are representative of three independent experiments.

We next investigated whether a cellular immune response was induced in animals immunized with the pcTPANS1 DNA vaccine. The presence of activated T cell subpopulations was analyzed in spleen and blood samples of vaccinated and control animals (non-immunized or pcTPA-inoculated mice), before and after virus challenge. Activation was determined by the expression of CD45RB^low^ on cell surface. No significant difference in the percentage of activated CD4^+^ or CD8^+^ T cells was observed among all non-infected mouse groups. On the other hand, we found that the percentages of both TCD4^+^CD45RB^low^ and TCD8^+^CD45RB^low^ significantly increased in samples of vaccinated animals after challenge, when compared to control groups (naïve or pcTPA-inoculated mice after infection) (Fig 5). Thus, these results suggested that the pcTPANS1 vaccine induced immune responses involving both CD4^+^ and CD8^+^ cells.

**Fig 6:**
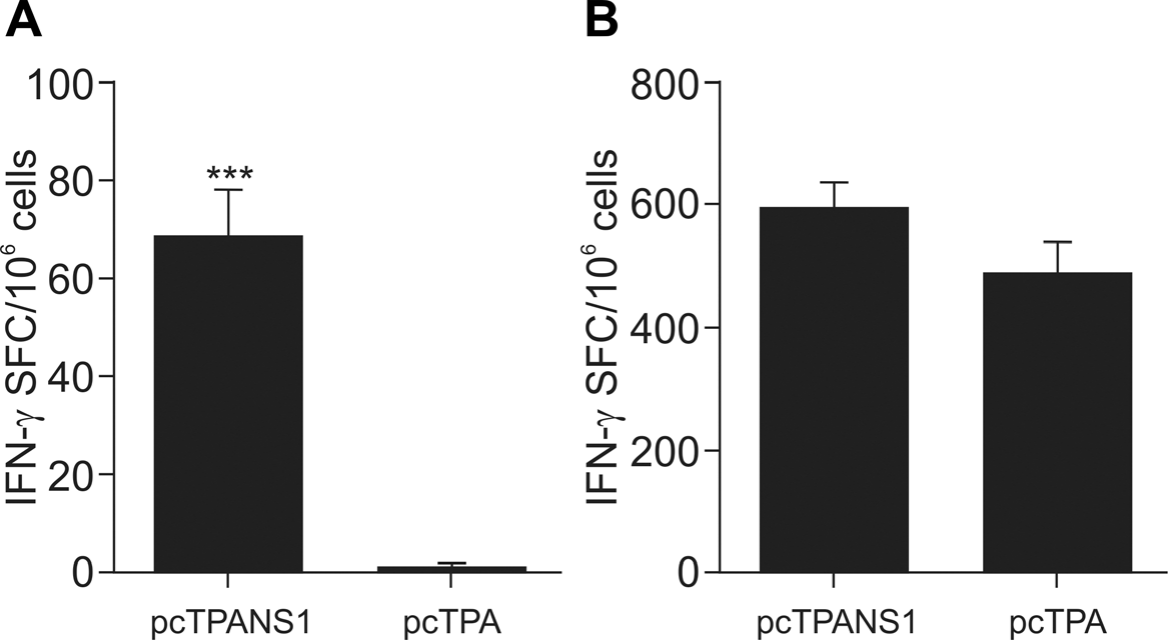
Production of IFN-γ by splenocytes from pcTPANS1-immunized mice. Spleens obtained from Balb/c mice inoculated with pcTPANS1 or pcTPA (n = 5) were collected 2 weeks after the second DNA dose and processed for IFN-γ ELISPOT assay. (**A**) Splenocytes were stimulated with the NS1 synthetic peptide ^265^AGPWHLGKL^273^ or (**B**) with concavalin A as a positive control. Numbers of spot-forming cells were quantified 24h after stimulation. Values are expressed as mean of IFN-γ spot forming cells (SFC) and standard error of the mean. Asterisks indicate statistically significant difference using Mann-Whitney test. *** p<0.001.

We then characterized some aspects of this T cell response using one of the few DENV-NS1 peptides described in literature as specific for Balb/c mice. Splenocytes from vaccinated or control animals were *in vitro* stimulated with the DENV-NS1 peptide (^265^AGPWHLGKL^273^), described as specific for CD8^+^ T cells, in an ELISPOT assay for detection of IFNγ production. Vaccinated animals presented significantly higher numbers of IFNγ-producing cells when compared to samples collected from pcTPA-inoculated mice (Fig 6A). Positive control using ConA as a mitogen confirmed the cell viability of all samples (Fig 6B).

**Fig 7:**
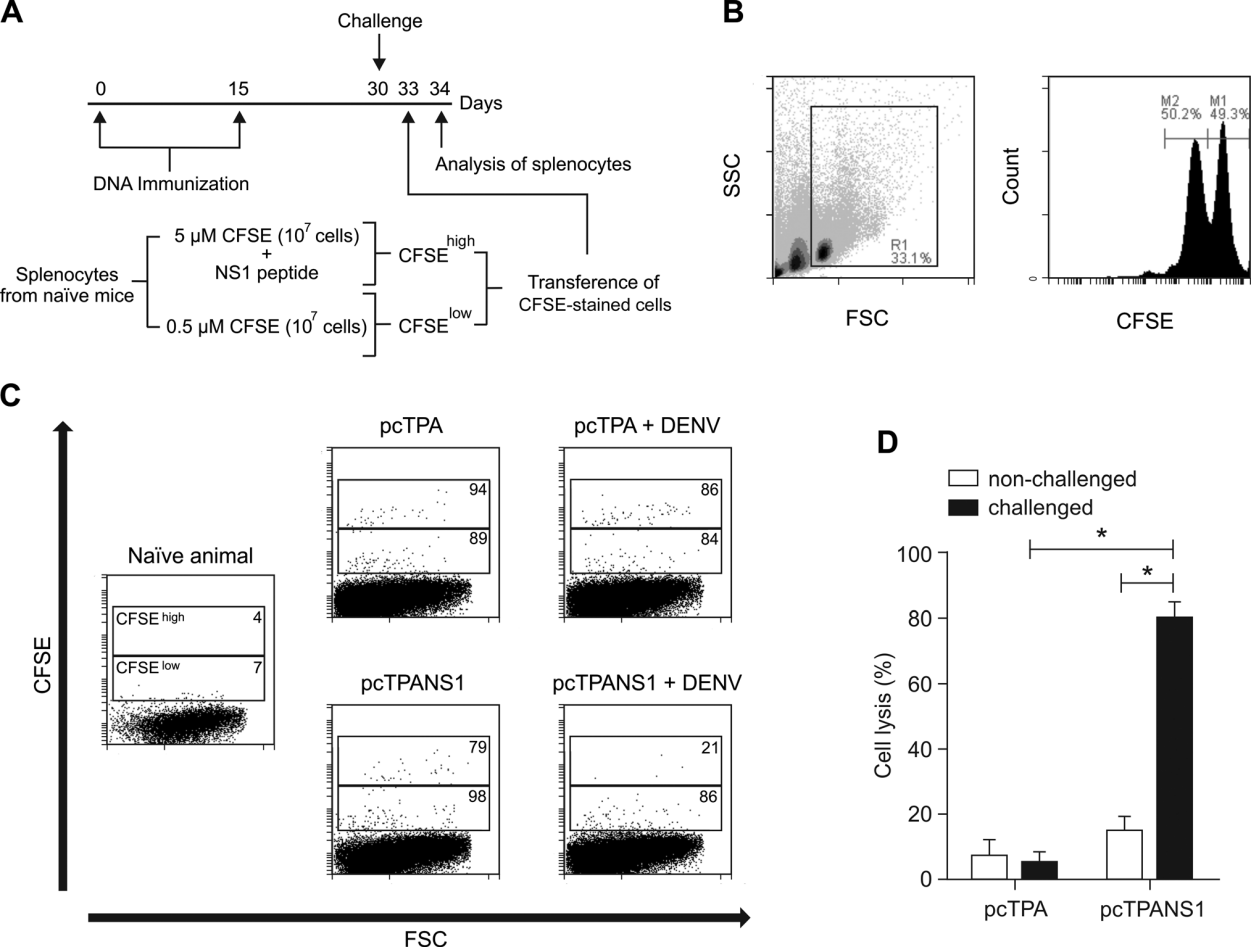
*In vivo* cytotoxicity assay. (**A**) Schematic representation of the assay. Balb/c mice inoculated with pcTPANS1 or pcTPA plasmids challenged or not with DENV2 (40 LD_50_) received a mixture of splenocytes obtained from naïve mice incubated with CFSE 0.5 μM or 5 μM (CFSE^low^ and CFSE^high^, respectively). CFSE^high^ splenocytes were previously pulsed *in vitro* with the synthetic peptide ^265^AGPWHLGKL^273^. Animals (n = 6) were sacrificed 20h after cell transference and splenocytes were collected and analyzed by flow cytometry. (**B**) Cytometric dot plot and histogram representing the mixture (1:1) of CFSE^high^ (M1) and CFSE^low^ (M2) splenocytes (inside R1) used for cell transference. (**C**) Representative dot plots of CFSE^high^ and CFSE^low^ splenocytes (top and bottom regions respectively) observed in recipient mice 20h after cell transference. Values represent the number of CFSE positive cells normalized to 20,000 considered events. (**D**) Percentages of cell lysis observed in analyzed groups calculated as follows: Cell lysis (%) = (1—CFSE^high^/CFSE^low^) x 100. Data are expressed as mean and standard error of the mean. Asterisks indicate statistically significant differences using Mann-Whitney test.** p<0.01.

**Fig 8:**
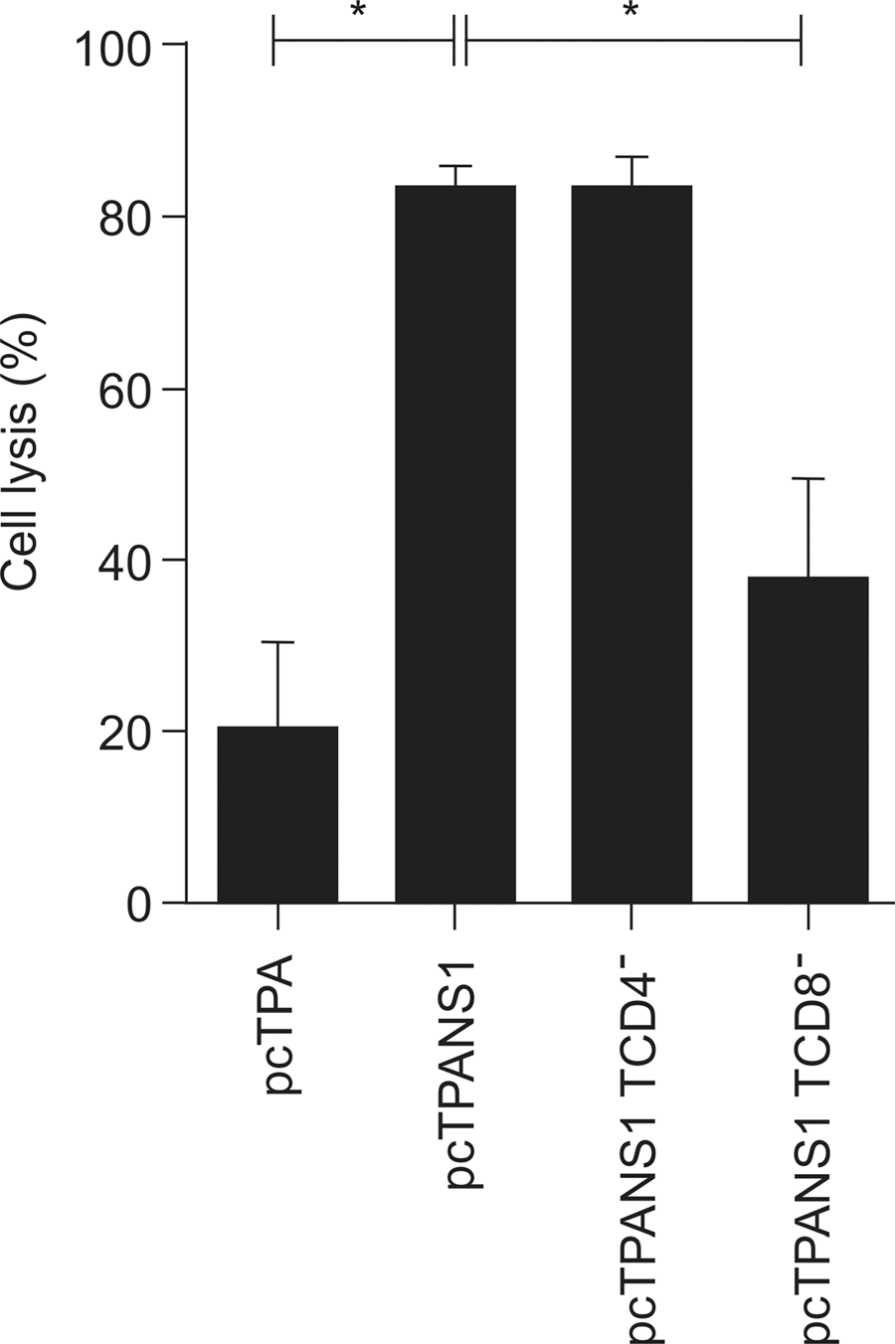
*In vivo* cytotoxicity assay with T cell depleted recipients challenged with DENV2. The *in vivo* citotoxicity assay was carried out as described in Fig 7 and groups of recipient mice (n = 6) were depleted from CD8^+^ or CD4^+^ T cells by inoculation of anti-CD4 or anti-CD8 antibodies, respectively. Values represent percentages of cell lysis as described in Fig 7, expressed as mean and standard error of the mean. Asterisks indicate statistically significant differences between groups Mann-Whitney test. * p<0.05.

To assess the functional activity of cells responding specifically to this DENV-NS1 peptide, we analyzed its cytolytic activity using an *in vivo* cytotoxicity assay in pcTPANS1-vaccinated animals, submitted or not to virus challenge (Fig 7A). Splenocytes isolated from naïve mice were pulsed with the DENV-NS1 peptide and labeled with high concentration of CFSE. Non-pulsed spleen cells, stained with low concentration of CFSE, were used to control non-specific cytolytic activity. Both cells, CFSE^high^ and CFSE^low^, were mixed (Fig 7B) and administered in vaccinated or pcTPA-inoculated mice three days after virus challenge. On the next day, animals were sacrificed and splenocytes were analyzed for detection of cell lysis, comparing high and low CFSE fluorescence intensity (Fig 7C). We observed only discrete cell lysis in vaccinated animals without virus challenge, while lysis increased significantly when pcTPANS1-immunized mice were challenged with DENV2 (5-fold higher when compared to non-infected animals) (Fig 7D). The cell lysis percentage in pcTPA-inoculated control animals, revealing non-specific activities, did not change after virus infection (Fig 7D). When a similar experiment of cytotoxicity assay was performed with vaccinated mice depleted from CD4^+^ or CD8^+^ T cells, we observed a significant decrease in cell lysis only after depletion of CD8^+^ T cells. No significant difference was detected between non-depleted or CD4^+^-depleted vaccinated animals, as well as between control pcTPA-inoculated mice and vaccinated group depleted from CD8^+^ cells (Fig 8). Thus, results confirmed the specificity of the DENV-NS1 peptide to CD8^+^ lymphocytes with no involvement of CD4^+^ cells.

### Contribution of T cells in the protection induced by the pcTPANS1 DNA vaccine

**Fig 9:**
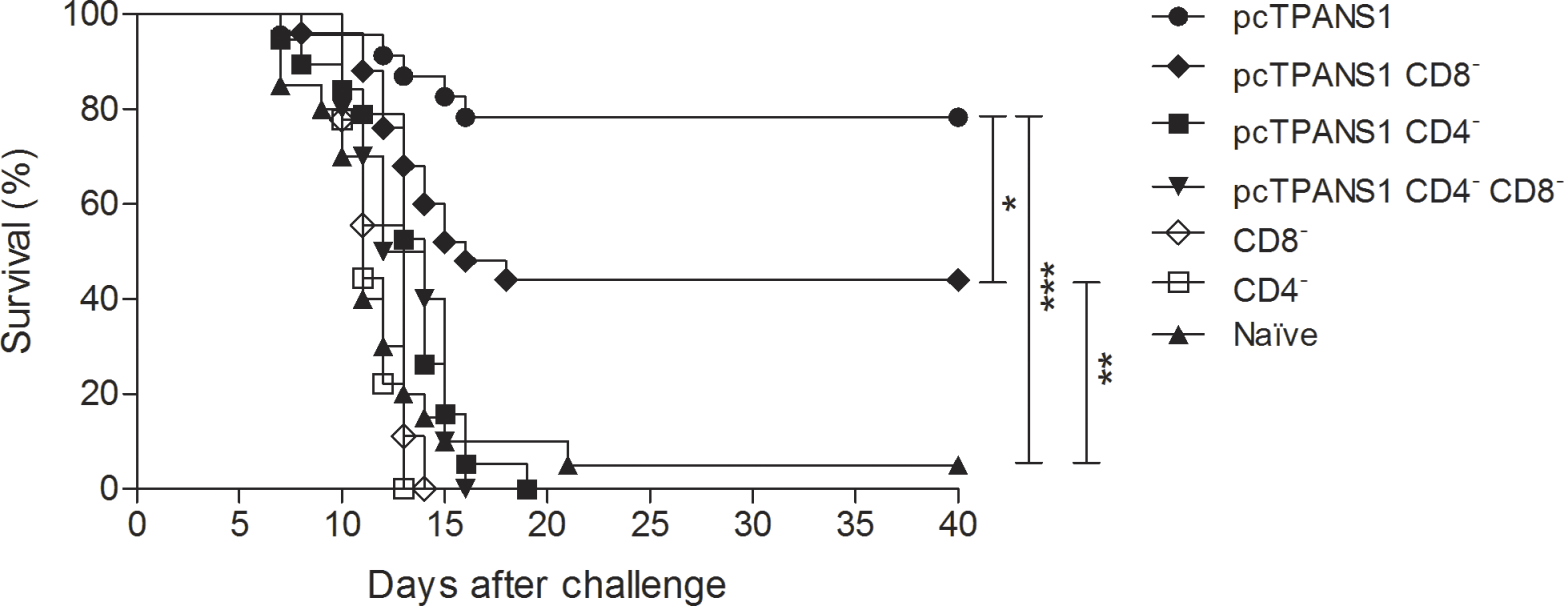
Survival rates of T cell depleted pcTPANS1-immunized mice after challenge with DENV2. Balb/c mice immunized with pcTPANS1 (n = 10) were intraperitoneally injected with 3 doses of anti-CD4 or anti-CD8 ascitic fluid before virus challenge (40 LD_50_) and one dose after infection. Controls included non-immunized or vaccinated mice challenged with DENV2. Animals were monitored for 40 days after challenge. Data represent compilation of three independent experiments. Asterisks indicate significant differences using Log-Rank statistical test. ***p<0.001.

In order to examine the role of CD4^+^ and CD8^+^ cells in the protection conferred by the pcTPANS1 vaccine, mice were submitted to antibody treatment for depletion of these cells, before and after virus challenge. Previously, the depletion procedure was standardized by several inoculations of ascitic fluid containing antibodies against CD4 or CD8, which yielded more than 99% reduction of such cells in mouse blood samples (S1 Fig and S2 Table). Depletion of CD8^+^cells reduced survival rates from approximately 80% to 45% of vaccinated animals challenged with DENV2 40 LD_50_ (Fig 9). Interestingly, all pcTPANS1-inoculated mice depleted from CD4^+^ cells died after virus infection (Fig 9). As expected, none of vaccinated animals depleted simultaneously from CD4^+^ and CD8^+^ cells survived virus challenge. Naïve control mice depleted from CD4^+^ or CD8^+^ also succumbed infection.

**Fig 10:**
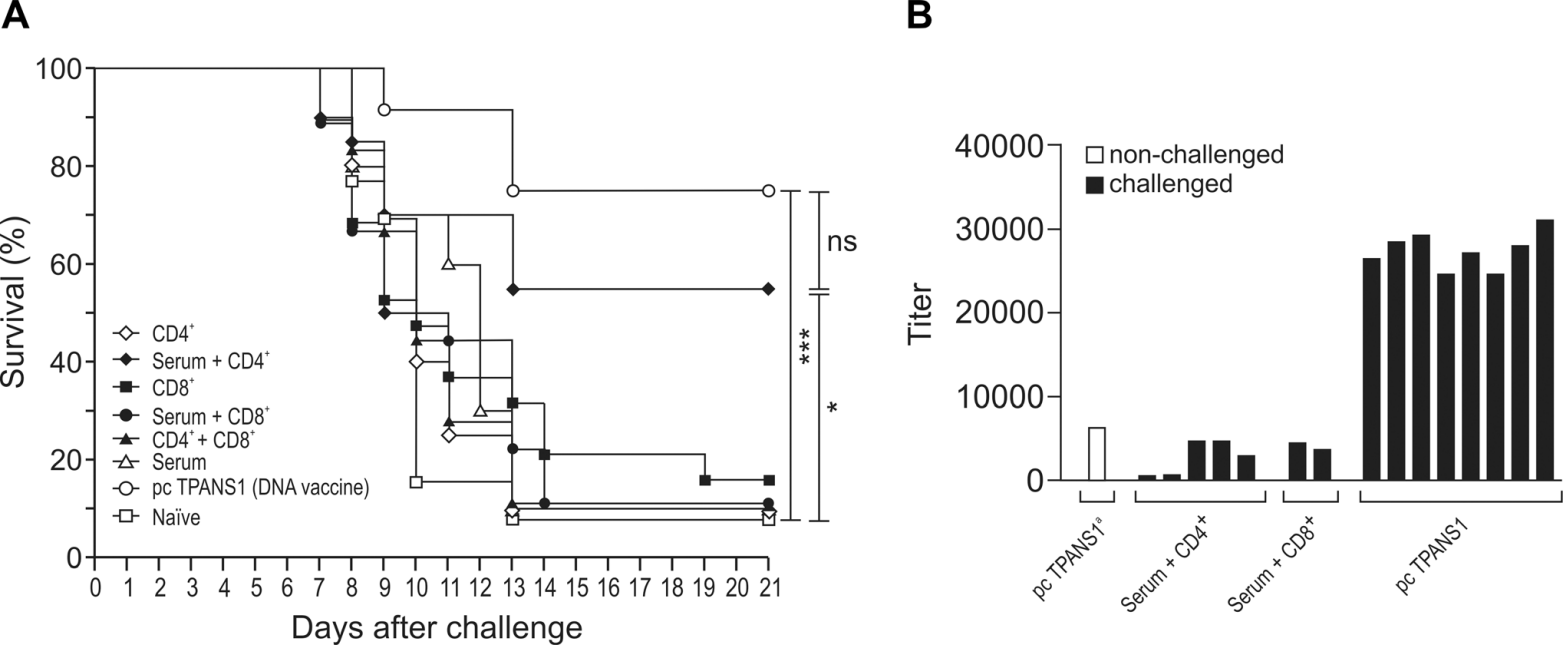
Protection induced by T cell adoptive transfer and antibody response (A) Survival rates of Balb/c mice after T cell adoptive transfer and challenge with DENV2. Enriched CD4^+^ or CD8^+^ T cell populations isolated from splenocytes of pcTPANS1-immunized mice were intravenously transferred to naïve Balb/c recipients. Animals were inoculated only with isolated cells or together with pooled sera obtained from pcTPANS1-immunized mice (one dose intraperitoneally injected). One day after cell transference mice were challenged with DENV2 (40 LD_50_) and monitored for the next 21 days. Data represent a compilation of two independent experiments with groups of 10 animals in each test (n = 20). Asterisks indicate significant differences using Log-Rank statistical test. ns (non-significant); *p<0.05; ***p<0.001. (B) Individual NS1-specific antibody response in serum samples collected from survived animals of one representative experiment evaluated by ELISA. Anti-NS1 antibody was also assessed in pooled sera (pcTPANS1^a^) used for inoculations.

We subsequently evaluated the influence of CD4^+^ and CD8^+^ T lymphocytes in the protection against DENV2 by adoptive transfer of these cells collected from pcTPANS1-vaccinated animals (without virus challenge). The procedure for enrichment of CD4^+^ and CD8^+^ T cells was previously standardized by negative selection using specific antibodies. In the CD4^+^ enriched population, we detected a reduction of approximately 85% of CD8^+^ cells, while the CD8^+^ enriched population presented almost a 100% depletion of CD4^+^ lymphocytes. All populations exhibited depletion of 90% of B220^+^ cells (S3 Table). For the adoptive transfer immunization procedure, we also included groups of animals that received cells and one dose of 500 μl of pcTPANS1-immunized mouse serum, simultaneously. Surprisingly, we only observed protection after challenge with DENV2 40 LD_50_ in the mouse group that received CD4^+^ T cells together with serum from vaccinated animals. In fact, 55% of animals in this group survived virus infection, which was not significantly different from the group of vaccinated mice (Fig 10A). All other tested groups, including those receiving CD8^+^ T lymphocytes together with serum or CD4^+^ and/or CD8^+^ T cells alone, were not significantly protected (Fig 10A).

The NS1-specific antibody response was analyzed in serum samples of animals that survived challenge after the adoptive T cell/serum transfer experiment. As expected, mice receiving T cells and serum did not presented a significant increase of anti-NS1 antibody titers, analyzed 21 days after virus challenge, thus confirming the absence of previous B cells primed by this antigen (Fig 10B). On the other hand, all pcTPANS1-vaccinated animals showed a boost of the humoral immune response, with a remarkable increase of anti-NS1 antibody levels (Fig 10B).

## Discussion

In this report we investigated the contribution of the humoral and cellular immune responses induced by a DNA vaccine (pcTPANS1) encoding the NS1 protein in Balb/c mice challenged with DENV2. We observed that both responses were important for protection against dengue. In fact, results revealed that an effective protection against virus challenge was not achieved with antibody or T cells only, but rather with the combination of both responses.

DNA immunization may be an interesting approach for the development of a vaccine against dengue, since it is recognized as a useful tool to induce both arms of the immune system. Different DNA vaccines have been tested against DENV, most of them based on the dengue virus envelope protein [47–49]. However, several reports have shown that the E protein is also involved in the phenomena of antibody dependent enhancement (ADE), where instead of protection by neutralization, antibodies against this protein may lead to an increase of virus replication [50–52]. Thus, the use of NS1 as antigen may be an alternative, since this protein is not involved in the ADE. On the other hand, other studies suggested an association between the immune response elicited by the NS1 and the pathogenesis of dengue with the generation of auto-antibodies. This fact was claimed to be important in regard to the damage effects observed in DENV infections, although the precise mechanism that lies behind it is not fully understood [27–34]. Anti-NS1 antibodies were shown to cross-react with elements such as platelets, fibrinogen and hepatic endothelial cells, also leading to increased serum levels of ALT and AST [27,31,34,53]. However, there is an apparent paradox between the disease recovery and high levels of anti-NS1 antibodies detected in convalescent patients [18–20]. In the present work, besides mapping the protective immune response elicited by the plasmid pcTPANS1, we also investigated whether this vaccine induces hepatotoxicity in immunized mice. Histological analysis showed no parenchyma or vascular damages in the hepatic tissue of these animals. Furthermore, vaccinated mice presented serum levels of the liver enzymes ALT and AST similar to control animals (pcTPA-inoculated mice), thus confirming preserved hepatic function. Therefore, our results suggested that the *in vivo* expression of the NS1 mediated by the pcTPANS1 vaccine, and consequently the immune response elicited against it, is safe without noticeable pathogenic effects in this mouse model.

We have previously shown that mice immunized with pcTPANS1 presented antibodies that recognized mainly conformational epitopes in the NS1 protein [23]. It is known that secretion of the recombinant protein mediated by DNA vaccines is crucial for induction of an effective humoral immune response [54–56]. In this regard, we have shown before that secretion of the recombinant protein due to the t-PA signal sequence, encoded by the pcTPANS1, was more efficient to generate protection against DENV when compared to the NS1 native signal peptide (present on the C-terminal region of E protein) encoded in another DNA vaccine [24]. In the present study, we confirmed protection yielded by the pcTPANS1 against DENV2. Besides, we observed that a 10-fold increase of viral LD_50_ (from 4 to 40) did not impact significantly on the survival rate of vaccinated animals challenged with DENV2, thus highlighting a robust protective immunity induced by this DNA vaccine. Furthermore, animals also survived virus infection when challenge was given one month after the last DNA dose, suggesting a long-term protection. Unfortunately, mice could not be challenged after a long time post immunization because aged animals became resistant to virus infection.

We next focused our efforts on the investigation of components of the immune response that are involved in protection elicited by the pcTPANS1. Levels of anti-NS1 antibodies increased considerably after virus challenge, characterizing memory and booster response after the secondary exposure to the antigen. We observed that passive immunization with several doses of anti-NS1 antiserum, obtained from pcTPANS1-inoculated mice, yielded partial protection against a lethal challenge with DENV2. These data corroborate with the literature regarding dengue [21] and other flaviviruses, such as yellow fever [57] and Japanese encephalitis virus [58], in which anti-NS1 polyclonal serum transfer seemed to confer a limited defense against lethal virus dose. In addition, we found that this protection was completely abrogated when mice were challenged with a 10-fold higher viral dose (40 LD_50_), thus suggesting that only anti-NS1 antibodies were not able to control infection with high viral load.

In regard to the cellular immune response induced by pcTPANS1, we observed activation of both CD4^+^ and CD8^+^ T cells, identified by low expression of CD45RB on cell surface. This response was significantly detected only after virus inoculation. This seems to be the ideal situation for a vaccine, where an exacerbated response without the presence of the pathogen is not desirable. In sequence, we found that vaccination with pcTPANS1 induced a cellular immune response against to the DENV-NS1 peptide ^265^AGPWHLGKL^273^, described as specific for CD8^+^ T cells (45). We noted that splenocytes isolated from vaccinated animals produced IFN-γ after *in vitro* stimulation, demonstrating the potential of a CD8^+^ T cell induction after vaccination. Under *in vivo* conditions, we also detected a T cell cytotoxic activity directed to the same peptide. The *in vivo* cytotoxicity was observed mainly after virus challenge, in accordance to results discussed above regarding detection of T cell activation. This activity was significantly abolished when mice were depleted from CD8^+^ T lymphocytes, thus confirming the specificity of the peptide used in this assay, with no participation of CD4^+^ cells.

Studies with rhesus macaques also pointed that NS1-specific CD8^+^ T cells are activated in dengue infection, with the production of IFN-γ [59]. Part of these cells was positive to CD107a on their surface, which is a degranulation marker and indicates cytotoxic activity [59]. Moreover, other reports have also demonstrated the participation of CD8^+^ T cell response in protection against dengue with a correlation between its cytotoxic activity and secretion of IFN-γ, which contributes to viral clearance [60]. However, in the study of Yauch *et al*. [60] the cell response was not directed to the NS1. Our initial results also suggested that CD8^+^ T cells would play an important role in the protection mediated by the pcTPANS1 DNA vaccine. However, in our next set of experiments we observed a predominant response of CD4^+^ T cells in the protection conferred by the pcTPANS1.

In fact, approximately 45% of animals immunized with the pcTPANS1 and depleted from CD8^+^ cells survived virus challenge, while all vaccinated animals depleted from CD4^+^ cells died after infection. Yauch *et al*. [61] also showed that CD4^+^ T cells are important for viral clearance. Authors suggested that CD8^+^ T cells play an important protective role in primary dengue infection, while CD4^+^ T cells are essential in the secondary response, which would be fundamental for vaccination [60,61]. One possible reason for such importance in secondary response would be a helper activity of CD4^+^ T cells for activation of B and/or CD8^+^ T lymphocytes. However, we found that transfer of CD4^+^ together with CD8^+^ enriched T cell populations from vaccinated mice was not protective in animals challenged with DENV2 40 LD_50_. Thus, results exclude the hypothesis that the helper function of CD4^+^ over CD8^+^ T cells would represent the major mechanism involved in the protection here conferred by CD4+ lymphocytes induced by the pcTPANS1. On the other hand, several animals receiving enriched CD4^+^T cell population together with anti-NS1 antiserum, both obtained from vaccinated mice without virus challenge, survived dengue infection. Besides, survival rate in this animal group did not significantly differ from pcTPANS1-immunized mice. Furthermore, most of animals that received only one dose of anti-NS1 antiserum did not survive virus challenge. It is important to emphasize that, although enriched CD4^+^ or CD8^+^ T cell populations still contained other cells (not CD4^+^, CD8^+^ or B cells), these contaminants were present in both enriched populations and, therefore, seem not to interfere with results demonstrating the importance of the CD4^+^ T cells in the protection elicited by the pcTPANS1. As expected, no significant increase in NS1-specific serum antibody levels was detected in animals that received CD4^+^ cells together with anti-NS1 antiserum. These data indicated that the protection observed by the transfer of pcTPANS1-elicited CD4^+^ T cells is not because of a helper activity of these cells over B lymphocytes, which would be also transferred from pcTPANS1-vaccinated mice as a contaminant leading to a booster of the humoral immune response.

In the present study we used the experimental murine model of Balb/c challenged intracerebrally with a brain mouse adapted DENV2 to test and map the protective immune response induced by the pcTPANS1 DNA vaccine. Unfortunately, there is no immunocompetent murine model that can mimic all the disease spectrum of dengue as observed in humans. We chose this approach since our main goal was to study the immune response elicited by our vaccine and this is an immunocompetent mouse model available. Besides, this model has been widely used for vaccines tests against dengue virus [62–67]. Although the symptoms manifested in this model are not exactly the same as described in humans, it has been reported that dengue infection can also lead to encephalitis in some fatal cases. Moreover, detection of viral antigens or dengue RNA was also observed in the brain of these patients [68–70]. Such evidences indicate the involvement of the central nervous system in the pathogenesis of dengue. On the other hand, in the chosen mouse approach not all animals died after showing hind leg paralysis and/or alteration of spinal cord. Approximately 35% of mice succumbed to infection without exhibiting morbidity. We still do not know the exact cause of death in these animals but viremia was detected in most of naïve animals after the DENV i.c. inoculation, indicating that virus can spread systemically, which may also contribute to death.

To conclude, we understand that the robust protective immunity generated by the pcTPANS1 in mice is strongly given by CD4^+^ T cells and the presence of antibodies, although the CD8^+^ T cell response may also contribute for protection. The mechanism involved in this protection is yet to be elucidated and further studies will be necessary to clarify this issue.

## Supporting Information

S1 Fig Depletion of T cells in Balb/c mice. Representative cytometric dot plots showing percentages of TCD4^+^ and TCD8^+^ cells observed in peripheral blood of naïve or T cell-depleted Balb/c mice. Anti-CD8 or anti-CD4 antibodies were intraperitonially administered for depletion of lymphocyte populations.
(TIF)

S1 Table Virus detection in serum samples of naïve and vaccinated mice infected with DENV2. (XLSX)

S2 Table Percentage of CD4^+^ and CD8^+^ cells in depleted Balb/c mice. (XLSX)

S3 Table Percentage of CD4^+^ and TCD8^+^T cells in enriched populations from Balb/c mice. (XLSX)
