# Supplementary figures and images for: Cooperation between CD4+ T Cells and Humoral Immunity Is Critical for Protection against Dengue Using a DNA Vaccine Based on the NS1 Antigen
Source: PLoS Negl Trop Dis. 2015 Dec 9;9(12):e0004277. doi: 10.1371/journal.pntd.0004277 (PMC4674122; doi:10.1371/journal.pntd.0004277)

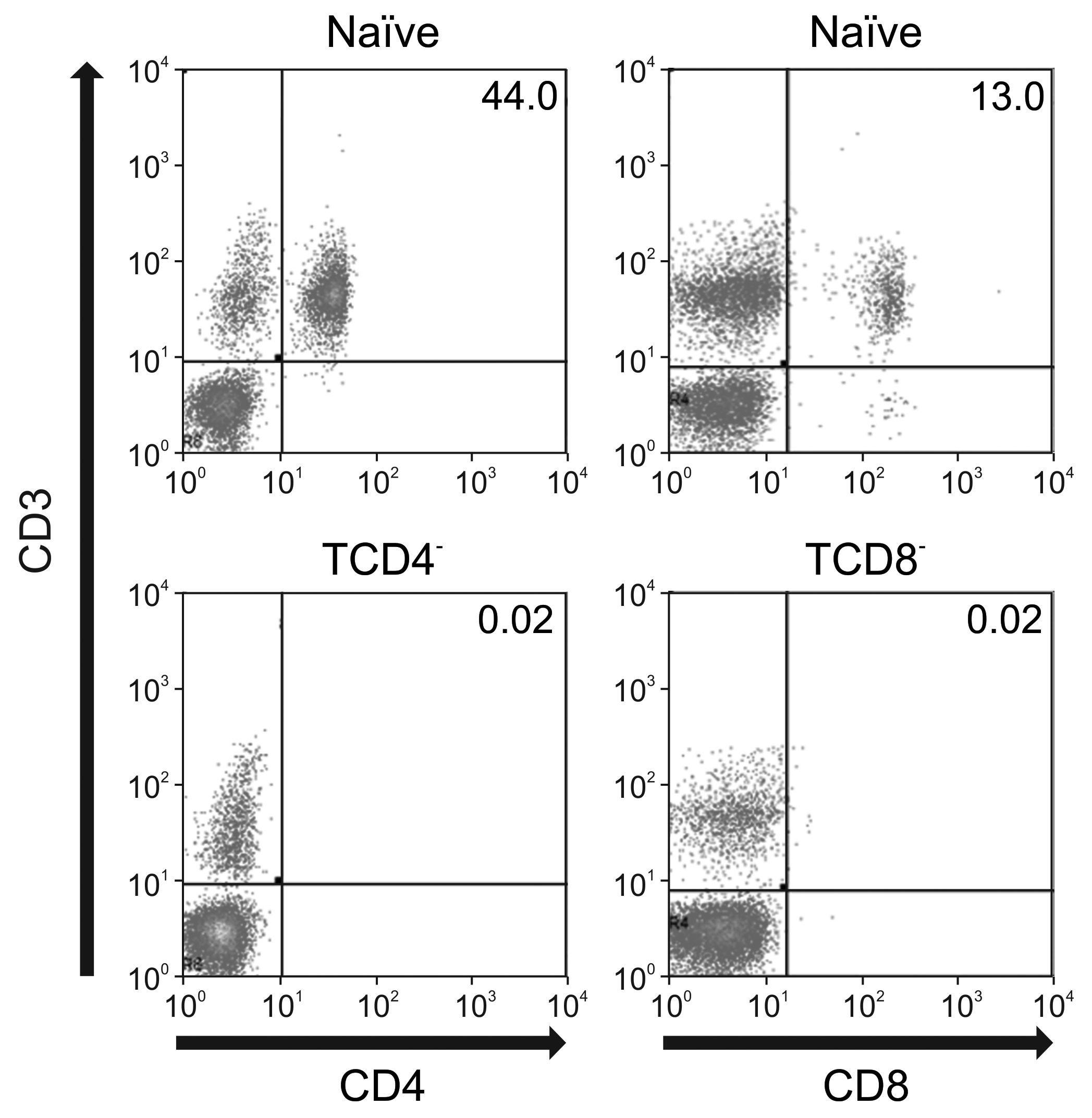

Supplement: S1 Fig — Representative cytometric dot plots showing percentages of TCD4+ and TCD8+ cells observed in peripheral blood of naïve or T cell-depleted Balb/c mice. Anti-CD8 or anti-CD4 antibodies were intraperitonially administered for depletion of lymphocyte populations. (TIF) [file pntd.0004277.s001.tif]
